# Stewardship and Shareholder Engagement in Germany

**DOI:** 10.1007/s40804-020-00195-8

**Published:** 2020-09-23

**Authors:** Wolf-Georg Ringe

**Affiliations:** 1 Professor of Law & Finance, University of Hamburg, Institute of Law & Economics, Hamburg, Germany; 2 Visiting Professor, University of Oxford, Faculty of Law, Oxford, UK

**Keywords:** Stewardship, Shareholder engagement, Institutional investors, Germany, COVID-19

## Abstract

Corporate stewardship holds great promise for the improvement of shareholder engagement and the encouragement of more responsible and long-term oriented value creation. This is particularly true since the outbreak of the global COVID-19 pandemic. Many countries have long adopted a best practice code for the stewardship role of institutional investors and asset managers, but Germany has so far refused to follow that trend. This paper explores the reasons for this reluctance, as well as whether the adoption of a Stewardship Code would still make sense in the regulatory framework of Germany today. Despite the increased presence of shareholder engagement (and even activism), several reasons may be put forward for why lawmakers have refused to adopt a stewardship code. This paper argues that the main political reason for this reluctance lies in the limited geographical reach of such a code, which would primarily be restricted to the (limited) domestic fund industry and would thus be unable to prescribe any meaningful principles to foreign-based asset managers. Still, I argue that the adoption of a code in the German context may make sense, for example to define expectations and to clarify the obligations of investee companies. Most importantly, it would benefit domestic investors that are typically ‘home biased’ and thereby frequently disproportionately invested in domestic funds.

## Introduction

Shareholder engagement is one of the most significant issues in corporate governance today. Ever since regulators identified passive shareholders, or ‘absent owners’, as one of the key governance problems that contributed to excessive risk-taking leading up to the financial crisis, regulators have been busy designing ways to improve shareholder ‘engagement’. The goal is to promote shareholder engagement with their investee companies, and to encourage more responsible and long-term oriented value creation.^1^

The iconic ‘Stewardship Code’ in the UK is the most visible example of such activity. The UK Code sets forth a number of best-practice principles that institutional investors, asset managers and proxy advisors are expected to follow. It was originally adopted in 2010 by a body known as the Financial Reporting Council (FRC), and is directed at asset managers who hold voting rights in UK firms. The principal aim of the Code is to encourage institutional investors, who manage ‘other people’s money’, to pursue a more active and engaged attitude towards their investee firms’ corporate governance. The idea of releasing such a stewardship code has been followed around the world, and some 25 jurisdictions now have one. Most recently, the key concept of adopting best practices guidelines for institutional investors has found its way into EU legislation, in particular the revised Shareholder Rights Directive 2019 (SRD II).^2^

Yet the EU’s largest economy remains surprisingly reluctant to join in the merry go-round. Lawmakers and regulators in Germany have been sitting on the fence on the issue during the past several years, hesitating on what to do. Most saliently, Germany has refused to adopt an official stewardship code, and the SRD II reform is eyed with some suspicion. This paper explores the role that stewardship and shareholder engagement play in the German context and investigates the reasons behind the reluctance of lawmakers to follow the international trend.

As we shall see below, although shareholder engagement is awakening amongst German investors recently, regulators have refused to develop a code mandating stewardship or shareholder engagement. While many doctrinal or functional reasons are put forward to explain this, this paper argues that the main political reason for this reluctance lies in the limited geographical reach of such a code, which would primarily apply to the (limited) domestic fund industry and would be unable to prescribe any meaningful principles to foreign-based asset managers. Still, I argue that the adoption of a code in the German context may make sense, for example to define expectations and to clarify the obligations of investee companies. Most importantly, it would benefit domestic investors that are typically ‘home biased’ and thereby frequently disproportionately invested in domestic funds.

This paper is organised as follows: Sect. 2 discusses the emergence of ‘stewardship’ as a phenomenon and traces its development from being a post-crisis shareholder engagement remedy to the much broader, ESG-encompassing silver bullet of today’s equity markets. Section 3 then turns to the question of why Germany has so far refused to give stewardship any regulatory backing. Section 4 discusses whether, despite the ostensible difficulties, the introduction of a stewardship code would still be desirable and concludes that an additional useful scope for it remains. Section 5 concludes.

## Stewardship and Shareholder Engagement

### Encouraging Shareholders

The roots of the current debate around increased shareholder engagement are to be found in the 2008 global financial crisis (GFC). Policymakers and academics identified many reasons behind the disaster, and one of the reasons put forward were severe flaws in the system of corporate governance. In particular, shareholders frequently got the blame. Many saw a lack of critical oversight by shareholders as the key problem, and institutional investors in particular were criticised for their ‘passivity’.^3^ Crucially, the seminal Walker Review of Corporate Governance in the UK Banking Industry, led by Sir David Walker, found in 2009 that institutional investors ‘appear to have been slow to act where issues of concern were identified […] and of limited effectiveness in seeking to address them either individually or collaboratively’.^4^ Further, the review stated that ‘the board and director shortcomings […] would have been tackled more effectively had there been more vigorous scrutiny and engagement by major investors acting as owners’.^5^ This claim was picked up by public figures such as the former UK City Minister Lord Paul Myners, who accused institutional investors of being ‘absentee landlords’.^6^ The subsequent Kay Review considered improvements to the UK equity markets and to long-term decision making, with a special focus on corporate and investor behaviour.^7^

It is against this background that the FRC was eventually charged with developing a specific instrument to improve shareholder engagement.^8^ Following a consultation in early 2010,^9^ the FRC was rather quick to publish the original UK Stewardship Code in July 2010^10^ along with a separate report concerning its implementation.^11^ The Code was revised in 2012^12^ and again in 2019.^13^

At the moment of its birth, the policy objective of stewardship was twofold. At an individual company level, stewardship was expected to help promote high standards of corporate governance and performance of the investee company. In other words, its role is a supportive one, to call upon corporate owners to fulfil their governance responsibilities. Here, stewardship is about enhancing long-term value creation. At the same time, stewardship should also operate on the investor level (i.e. between the investor and the asset manager).^14^ Reinforcing the accountability of institutional investors and asset managers to their clients should also strengthen trust in the financial system more generally.^15^

Initially, the use of the UK Stewardship Code had just been recommended on a voluntary basis. However, with effect from 6 December 2010, the then Financial Services Authority^16^ required that UK-approved asset management companies must disclose to what extent they comply with or derogate from its requirements.^17^ For other institutional investors and foreign investors whose investment activity extends to companies in the United Kingdom, a non-binding recommendation for use remains without publicity.

A similar trend was soon picked up elsewhere. After the financial crisis, the European Commission also looked into corporate governance, resulting in a 2010 Green Paper.^18^ In this context, the Commission argued that ‘shareholders do not seem to have fulfilled their role of “responsible owners”’^19^ and that they ‘seem to show little interest in the long-term governance objectives of the businesses/financial institutions in which they invest’.^20^ Former Commissioner Michel Barnier said in a 2010 speech that ‘[w]e have spoken for years about shareholder rights. It is time to also talk about shareholders’ obligations’.^21^ Following the Green Paper consultation, the vast majority of respondents supported further legislative activity in this field.^22^ This concerned, in particular, institutional investors’ obligation to publish their voting policies and records. The hope was that public disclosure would improve investor awareness, optimise investment decisions by the ultimate investors, facilitate issuers’ dialogue with investors, and encourage shareholder engagement.

The European Commission first analysed these issues in the more specific context of corporate governance of banks and financial institutions, but has subsequently taken listed companies generally into consideration.^23^ A 2011 Green Paper thus subsequently extended the framework to public companies more generally and identified short-termism as the main factor contributing to market inefficiency. The measures discussed were addressed to institutional investors and included the publication of their voting decisions, the identification and publication of any conflicts of interest, the disclosure of a remuneration policy for financial intermediaries, and an improvement in the level of information to investors about the risks associated with an investment. An academic ‘Reflection Group’ also considered whether an EU-wide best practice code might be a useful tool.^24^

At the end of 2012, the Commission then presented an Action Plan, which transferred the theoretical framework into specific regulatory objectives for the coming years.^25^ The Action Plan was based on the three main areas of improving transparency between the company and its investors, strengthening the long-term commitment of shareholders, and improving the legal framework for the cross-border operation of firms.^26^ This ultimately led to the revision of the original 2007 Shareholder Rights Directive to introduce elements of the stewardship idea.^27^ It took however some time until this went ahead. The SRD II was adopted in May 2017, and Member States were obliged to implement the new standards by June 2019.^28^

In the meantime, the trend has led to the adoption of codes on active ownership in many countries—not just in the EU, but worldwide.^29^ International bodies such as the International Corporate Governance Network (ICGN) have also published their own standards.^30^ The OECD has also done work in this area^31^ and has incorporated stewardship elements into its Corporate Governance Principles.^32^ Industry bodies such as the European Fund and Asset Management Association (EFAMA) have also adopted their own stewardship rules.^33^

All these codes, best practice guidelines, and also the SRD II, share a number of common elements. Besides encouraging shareholder engagement, they typically require institutional investors to disclose their engagement policies, and the results of their implementation. Moreover, these instruments also emphasise that shareholders should play a more active role in ensuring that companies are accountable not only to shareholders but also to society as a whole.

### ESG Stewardship

So far, we have considered the genesis of stewardship as a shareholder-focused development, which was intended as a means to foster shareholder engagement and to monitor managerial excessive risk-taking. More recently, stewardship has morphed from this original purpose to cover a broader set of issues, which have become known as ‘ESG’ (Environment, Social and Governance) policies. This is an umbrella term for investment policies that seek positive returns and long-term impact on society, the environment and the performance of the business. With some granularity, the ESG agenda bears a certain resemblance to the well-established trend of Corporate Social Responsibility (CSR), which in turn has been rebranded and is now more commonly referred to as Socially Responsible Investing (SRI).^34^

From an investment perspective, ESG factors are a subset of non-financial performance indicators which include sustainable, ethical and corporate governance issues such as managing a company’s carbon footprint and ensuring there are systems in place to ensure accountability. They are factors incorporated into both investment decisions and risk management processes.

The ESG movement was boosted with the adoption of the United Nations Principles for Responsible Investment (UNPRI), a UN-backed set of principles, which aim at contributing to the development of a more sustainable global financial system.^35^ The PRI, originally launched in April 2006, received increased interest following the GFC and a sharp rise in the number of signatories.^36^ Further, the important proposal of an EU ‘framework for sustainable investment’ lists ten initiatives to strengthen financial stability through a stronger emphasis on ESG factors and to improve the contribution of the financial sector to sustainable growth.^37^ This instrument seeks to integrate ESG factors into the decision-making process of institutional investors and asset managers.^38^

Most recently, the ESG movement has found its way directly into stewardship principles. For example, the most recent version of the UK Stewardship Code, which came into force on 1 January 2020 recognises the importance of ESG factors: ‘The proposed Code now refers to environmental, social and governance (ESG) factors. Signatories are expected to take material ESG issues into account when fulfilling their stewardship responsibilities.’^39^ In a similar vein, the ICGN is currently consulting on a revised version of its Global Stewardship Principles. Among the various changes that are proposed, one of the key features includes the use of ESG factors in investment decision making, as well as in stewardship.^40^

Comparing this trend with the original policy objectives,^41^ it appears that the purpose of stewardship is expanded yet again: as we saw, it originally sought to fulfil a corporate governance purpose (at the investee company level) and an informed investment decision-making purpose (at the investor/investment fund level). Now, the investment approach includes the consideration of wider ethical, environmental and social factors and the consideration of relevant systemic risks. In a broader context, stewardship is thus seen as enhancing not only sustainability and long-term economic growth, but overall financial market stability.^42^

It is submitted that the move from a shareholder-oriented concept to an ESG programme changes the substance of stewardship considerably. Stewardship has always been hailed as the path to more sustainable investing and the path to a better world, but the inclusion of ESG standards gives the movement a quasi-religious authority. Seen in this light, adherence to ESG issues is frequently seen as the ‘holy grail’, with the potential of solving multiple problems of society at large. During the global COVID-19 pandemic, this trend has received even broader support worldwide. It is thus reminiscent of the general development of corporate governance, which has turned into a means of addressing all different social and economic issues that were once predominantly the concern of government regulation.^43^

The success story of stewardship and ESG principles thereby takes the place of lawmakers’ previous favourite governance feature: independent directors. As is well documented, many instances of corporate governance reform over the last several decades have seen an increased promotion of independence criteria and quotas.^44^ This is even more surprising given that academic evidence of their financial performance is mixed at best.^45^

Perhaps the latest trend towards shareholder empowerment and engagement is evidence of a learning process: it may be understood as regulators accepting the limited benefits of outside directors as a monitoring tool, and now identifying shareholders as the better incentivised group to take up a stronger governance role in the firm.

### Index Funds and Stewardship

Most recently, stewardship is facing fresh challenges due to market developments, notably the advent of index investing.^46^ Index funds are generally considered as a nightmare for corporate governance, since they do not take an interest in any strategic matters of their target firms; rather they only invest in a company because the company’s shares are part of an index. Despite these original fears, sceptical voices now see index funds in a more positive light. The reason is that index funds have no other option than to fix certain problems and to take measures that improve the performance of their portfolio companies, since exit is not a viable alternative for them.^47^ Accordingly, they have lately changed their voting patterns, and are more willing to vote together with activists—they are both becoming active owners^48^ and are particularly ESG-minded as their perspective is for the long run.

Exercising ‘voice’ instead of ‘exit’ creates a free-riding problem for the exercising investor.^49^ More specifically, an index fund’s performance is typically measured against the performance of rival index funds. If an index fund undertakes an investment in stewardship, this investment will increase the value of a particular portfolio company, but ‘the increase will be shared with all other investors in the company, including rival index funds that replicate the same index’.^50^ As a result, an index fund’s stewardship engagement offers competitive advantages to its rivals that share in engagement’s benefits without being subject to stewardship’s costs. Regulatory authorities have acknowledged this free-riding problem.^51^ The introduction of a regulatory initiative rendering stewardship a mandatory activity for all companies of the investment management industry can be perceived as eliminating the free-riding problem. However, such an introduction would face two challenges.

First, imposing an obligation to develop a stewardship activity is a dead letter if there are no guarantees about the quality of shareholder engagement.^52^ This argument was among the main concerns of industry stakeholders before the adoption of the UK Stewardship Code,^53^ and remains one of the main points of criticism against the effects of the Code.^54^ Market participants^55^ as well as regulatory authorities^56^ argue that less qualitative reporting of stewardship activities is prompted by a regulatory framework consisting of prescriptive mandatory rules.^57^ The need for more qualitative shareholder engagement explains the positive industry response to the introduction of an annual Activities and Outcomes Report besides the Policy and Practice Statement upon signing the Code.^58^

The second challenge has to do with the addressees of any regulatory initiative. Empirical evidence shows that even in jurisdictions where there is no regulatory initiative relating to stewardship, such as the US, asset managers integrate stewardship and ESG factors in their investment decision-making process. The most important fund managers are the ‘Big Three’, Black Rock, State Street Global Investors and Vanguard. Even though they do not have any legal obligation to perform stewardship, they do so on a voluntary basis. As such, the following question arises: What are the characteristics of these managers that incentivise them to invest in stewardship? The benefits they enjoy out of their stewardship activity must outweigh the costs they incur respectively.^59^

On the benefit side, the adoption of stewardship activities might have a positive impact on an institutional investors’ reputation, which leads to an increase of demand from end beneficiaries, provided that they have stewardship preferences and do select institutional investors based on these preferences. The same can be said for the selection of asset managers by institutional investors.^60^ On the costs side, empirical evidence supports the argument that the ‘Big Three’ underinvest in stewardship given the size of their portfolio.^61^ This underinvestment in stewardship in combination with the economies of scale that are achieved through the increase of common ownership held by the dominant institutional investors^62^ render the undertaking of stewardship activities by them cost-effective.

The first insight that we gain from the cost–benefit analysis of the integration of stewardship in the Big Three’s business model is that the economic incentive to undertake stewardship is associated with the size of the institutional investor or asset manager. This is confirmed by current literature^63^ and a qualitative analysis of market participants’ responses to the FRC 2010 public consultation.^64^ The differentiated cost-management between large and small institutional investors can transform stewardship into a regulatory barrier for small institutional investors and asset managers. For this reason, any regulatory initiative should be supplemented by initiatives that will create a level playing field in the investment management industry. Such an initiative can be, for example, the promotion of shared outside research services.^65^

## A Stewardship Code in Germany?

Having explored the general context in which the idea has developed, this section now moves on to consider stewardship and investor engagement in the German context particularly.

### Corporate Engagement in Germany

It is well documented that the German corporate ownership system has been undergoing profound changes over the past 20 years.^66^ Long seen as the paradigm example of an insider-dominated economy with strong networks across the country, the ownership of German firms has more recently opened up and given way to more outside holdings and more active engagement policies. Several studies^67^ have identified a constant increase of equity shareholdings in German companies held by institutional investors. Among them, investment fund management companies (KAGs) and insurance companies have the largest ownership stake. In contrast, German pension funds are not as developed as in other countries, such as the UK.^68^ The general increase can be attributed to legislative initiatives aiming to unwind cross holdings in German companies, such as the changes in capital gains taxation in 2001, the higher capital requirements for banks, and the implementation of new insider trading laws.^69^ The dissolution of cross-ownership has also been followed by a significant increase in the stake of foreign institutional investors in German companies.^70^ The presence of foreign investors is even more salient in DAX companies; meaning the German companies with the largest market capitalisation. Among the Top 15 DAX investors, the ‘Big Three’ have a very prominent position.^71^

Institutional investors’ corporate engagement activity is regulated by the German Stock Corporation Act (*Aktiengesetz*) and the Capital Investment Act (*Kapitalanlagegesetzbuch*).^72^ The first Act determines the rights and obligations of institutional investors in their capacity as shareholders, while the second Act determines their fiduciary duties towards their clients. Shareholder engagement entails a broad range of formal and informal types of corporate governance intervention.^73^ An example of formal shareholder engagement is the participation and the exercise of voting rights in Annual General Meetings (AGMs). Studies have shown that the average voting turnout rate in German companies fluctuates between 52 and 58%,^74^ which is low in comparison to the UK and other countries of continental Europe, such as France.

Informal shareholder engagement, such as behind-the-scenes communication between shareholders and the supervisory board of directors, can substitute the comparatively low turnout rate. This has been the case with hedge funds initiating activist campaigns in Germany.^75^ Hedge fund activists frequently employ informal means of communication with the board of directors before communicating their corporate governance concerns to the public.^76^ This escalation of shareholder engagement activities has also been adopted as a principle of the UK Stewardship Code 2020.^77^

All of this has led to more active engagement at companies’ general meetings.^78^ For example, many DAX executives and board members are likely to remember vividly the weak voting results at the 2016 and 2017 AGMs. After decades of approval rates beyond 90 percent, results with less than three-quarters of approval rate may not be read as an expression of fundamental mistrust, but rather as a clearer articulation of shareholder interests.^79^ This all culminated in the 2019 AGM of Bayer AG, where the shareholders, for the first time in German corporate history, refused to approve the management board (*Entlastung*).^80^ The reasons for this shareholder ‘revolt’ are manifold. Non-transparent compensation structures for management board members, blank authorisations for capital increases, and doubts about the independence of supervisory board members are the most frequent criticisms by shareholders.

The good news is that some companies apparently are listening and responding to the concerns of investors. For example, software company SAP has responded to the vote at the 2017 AGM and has made every effort to understand the scepticism of its shareholders in order to change the incentive structure and transparency of their compensation system.^81^ It appears that institutional investors are especially concerned about executive compensation issues, which is why rejection rates are highest for these corporate decisions.^82^ Other topics of concern include the capital structure, in particular the issuance of new shares or convertible bonds; there were doubts about the number of refusals in terms of independence of members of the Supervisory Board, and in particular members of the Audit Committee are subject to stringent requirements. Bayer was a special case: the public mistrust mostly stemmed from the disastrous performance of the share price since the firm had acquired US rival Monsanto, which resulted in mounting legal problems over glyphosate, a controversial weed-killer that may be causing cancer.^83^ These no-confidence votes are not legally binding and do not trigger any direct legal consequence. Still, the reputational damage for the management may be enormous: no member of the management or supervisory board can permanently afford to act against the will and without the trust of the shareholders.

### A Stewardship Code for Germany?

A number of explanations may be found that can contribute to the reluctance of German lawmakers to adopt a stewardship code. I will explore this question from a range of different perspectives. Most prominently, some academic commentators put forward a range of doctrinal objections to the project, arguing that a stewardship code would sit at odds with some key principles of German corporate law. One may, however, also understand the German position in more functional terms: since ownership here is more concentrated than elsewhere, stewardship may not be needed. A third account would rely on the presence of lobbying and interest group theory to explain objections against a stewardship code. Finally, and maybe most convincingly, there is a political dimension to the story. We shall explore these different explanations in turn.

#### Legal Objections

Academic commentators worldwide have long been critical towards the stewardship movement, citing many legal and doctrinal reasons of why such a concept would be alien to the German system of corporate law.

For example, many commentators in German academia respond to the very idea of stronger shareholder engagement and stewardship with outright hostility.^84^ They argue that increased shareholder engagement may interfere with the well-balanced system of checks and balances in German corporate governance.^85^ For example, a perceived ‘micromanagement’ of entrepreneurial decisions by investors would not be consistent with the management autonomy of the management board.^86^ In addition, they fear conflicts with the supervisory board’s supervisory responsibility as it is the supervisory board’s exclusive role to monitor management.^87^ In consequence, monitoring by institutional investors must under no circumstances lead to the establishment of a de facto ‘shadow supervisory board’.^88^ Further, an increased engagement of institutional investors may undermine the concept of shareholder equality, which is held dear in German doctrine.^89^ This principle demands equal treatment of shareholders with equal characteristics.^90^ It is argued the risk is that enhanced engagement leads to differentiated treatment of shareholders, for example by encouraging management to pass on confidential information only to certain (active) shareholders.^91^ However, if it can be shown that (some) institutional investors have specific characteristics that legitimise their differentiated treatment in comparison with other shareholders, the principle of equivalent treatment may not be violated.

In a similar vein, some commentators argue that the stewardship movement may grant institutional investors certain idiosyncratic ‘private benefits’ at the detriment of other shareholders.^92^ This may run against the well-established doctrine of fiduciary duties that shareholders are subject to, and may also be in conflict with German principles concerning corporate groups (the so-called *Konzernrecht*).^93^

Finally, a number of critics maintain that there is no good normative reason for why passive investment strategies, due to the ‘comply or explain’ mechanism, should bear the stigma of needing justification.^94^*Aktiengesetz* § 54(1)—the ‘*Magna Carta*’ of the shareholder—requires investors only to pay their financial contribution, and does not entail any other obligation; which is seen as significantly furthering the attractiveness of this form of investment.^95^ This principle is associated with the classical perception of shareholders as capital providers who do not bear any duties towards other shareholders, stakeholders, or the company per se.^96^ In the extreme, the share is seen as a piece of property that may be handled by its owner as they may please.^97^ This view would be difficult to reconcile with the sudden imposition of shareholders’ stewardship obligations. Still, it is widely accepted that the ‘no obligations’ rule in a pure form does not reflect reality. For example, it has been relaxed in favour of minority protection and market integrity.

More recently, in the context of the stewardship debate, the focus of attention has shifted to one particular aspect: the legal limits on a potential dialogue between institutional investors and the supervisory board.^98^ Scholars have pointed out barriers to such dialogue that may result from both corporate law and capital markets law. Corporate law barriers root once more in the perception of the supervisory board as an internal organ of the corporation with no or very limited external authority, and the division of authority between the management board and the supervisory board. Capital markets law barriers may arise from the insider trading prohibition and the limitations on ‘acting in concert’.^99^ This controversial issue was partly addressed in a 2017 reform of the German Corporate Governance Code (GCGC), which now states in Suggestion A.3 that the chairman of the supervisory board ‘should be available—within reasonable limits—to discuss Supervisory Board-related issues with investors’.^100^ This change goes back to proposals made by a working group composed of representatives of institutional investors, firms and academics, resulting in the adoption of guidelines for the dialogue between an investor and the supervisory board.^101^

#### Functional Story

In contrast to the doctrinal explanations sketched above, a better story for stewardship sceptics to tell might be that greater stewardship by institutional shareholders is not equally necessary in the German context, since the frequent presence of controlling shareholders (unlike in the UK) ensures that management is adequately monitored. This would be a *functional* argument: essentially, one could argue that the objective of the UK Code—to encourage greater investor engagement in the long-term—is not anything that needs to (or even should) be addressed in the German arena, given that the domestic nature of ownership concentration renders this objective superfluous.

This argument is, however, flawed on two grounds. First, the theoretical argument that a controlling shareholder may exercise close monitoring over corporate management has great appeal. After all, it has been argued that a controlling shareholder may police the management of public corporations effectively: because they hold a large equity stake, the argument runs, a controlling shareholder would be likely to have proper incentives either to monitor managers effectively or to manage the company itself and, because of proximity and lower information costs,^102^ may be able to detect any problems earlier.^103^ However, in reality, it has long been recognised that controlling shareholders bring their own problems with them. Controlling shareholders will frequently extract private benefits of control from the company, for example through a technique referred to as ‘tunnelling’, that is, through contractual dealings with the company, like transfer pricing, that favour the controlling shareholder.^104^ This (and many other techniques) is said to raise intra-shareholder agency costs where controlling shareholders may exercise power to the detriment of any minority investors. It is therefore an illusion to believe that stewardship in the German context may not be necessary just because the monitoring of corporate management would be carried out by any controlling shareholders.

Secondly, even when assuming for a moment that the first argument was correct, it has been demonstrated that the presence of controlling shareholders in German firms is shrinking. Since the turn of the century, triggered not only by globalisation forces but also by an idiosyncratic taxation reform in Germany, formerly large investors have started to divest of their equity holdings in domestic firms.^105^ This has triggered a fundamental rethink of the role of corporate law and corporate governance in Germany where the recognition is growing that the legal system must partly be reconfigured to cater for firms in dispersed ownership rather than being controlled.^106^

In light of these two considerations, it becomes evident that we cannot reasonably rely on controlling shareholders to exercise the role of a serious policeman in the German corporate landscape.

#### Economic Rationale

It is of course possible that lawmakers in Germany are simply not convinced that a stewardship code would have a meaningful impact. After all, the effectiveness of such a code is very difficult to measure, and a number of commentators have argued that there is no tangible benefit.

For example, the UK, as the frontrunner in terms of stewardship, has long seen sceptical comments on the stewardship concept.^107^ Many market participants are unconvinced that the British advance has actually resulted in increased shareholder engagement.^108^ However, most critical commentators fall short of providing reliable evidence for their claims. The 2018 Kingman Review into the operation of the FRC voiced some serious criticisms of the UK Stewardship Code.^109^ The Review recommended that a fundamental shift in approach would be needed to ensure that the Code more clearly differentiates excellence in stewardship. According to Kingman, the Code should focus on ‘outcomes and effectiveness’ of the stewardship process, and not on the formal policy statements.^110^

Edward Rock, in a recent article, put forward a rather disillusionist experience report from a 2003 ‘mutual fund experiment’ in the US.^111^ An SEC release from 2003 mandated US mutual funds disclose proxy voting policies and proxy votes, and described ‘best practices’ for proxy voting guidelines. Rock describes that the industry responded by turning the requested activities into ‘compliance function’, a rather schematic box-ticking exercise, and that investors apparently do not care about the disclosures.^112^

It is however unlikely that lawmakers would be unaware of more positive findings in the academic literature. For example, a study by Hoepner and co-authors has revealed that stewardship and promoting of ESG issues can have a positive impact on firm value and may also create value for other stakeholders.^113^ The authors conclude that companies with a completed ESG campaign have, on average, a significantly lower risk profile. Further, the authors were also able to show causation in a sense that the lower risk profiles result from successful stewardship activities, where a change was made to the respective company with regard to its ESG strategy. Another paper comes to a similar conclusion, showing that firms with a successful ESG engagement are followed by positive abnormal returns.^114^

Consistent with this perspective, other research has found that where institutional investors engage more deeply with their portfolio companies, these firms are more likely to pursue innovative strategies.^115^ Similarly, a 2017 study concluded that without the discipline of active engagement by investors, a company’s management is more likely to become entrenched and to engage in value-destroying M&A activity.^116^

Finally, and more generally, a recent article finds that the overall quality of the capital market has a strong impact on economic performance, supporting long-term sustainable economic growth, and reducing the risk of financial crises. In the authors’ view, market quality may be improved by greater transparency requirements and by promoting more active investor engagement.^117^

Where does this leave us? To be sure, scepticism is widespread, but may not always be founded on concrete evidence. There is, by contrast, growing academic literature that acknowledges and demonstrates the economic case for stewardship and shareholder engagement, especially with a focus on ESG policy. The argument that lawmakers ought to reject further stewardship initiatives with reference to their uncertain effects thus remains a hypothesis at best.

#### Political Perspective

Instead, probably the most convincing explanation for German lawmakers’ reluctance to subscribe to the stewardship idea seems to be rooted in politics. At its core, the reason is simple: as the market share of Germany-based institutional investors is small in comparison to other jurisdictions, it would not be an optimal allocation of public resources to devote legislative energy to a project that would not improve shareholder engagement in any meaningful way.

**Fig. 1:**
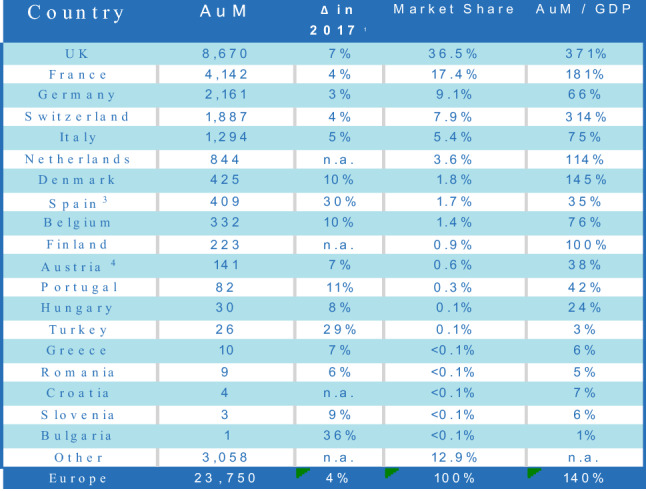
European AuM by geographical breakdown at end 2017 (in EUR billion and %). Source: EFAMA (2019), p 11

A few figures may illustrate the point (see Fig. 1). Germany is the third largest European asset management market measured by assets under management (AuM) (at EUR 2161 bn). Nonetheless, its market share of 9.1% of the European market is far less than its economic power and its population size; in fact it is comparatively significantly smaller than the respective market share of the two largest asset management markets, the UK (36.5% with EUR 8670 bn AuM) and France (17.4% with EUR 4142 bn AuM). Furthermore, assets under management in Germany constitute 66% of German GDP, a ratio well below the UK (371%), French (181%), and European (140%) AuM/GDP ratio. One reason advanced is that there are no tax benefits for long-term savings with mutual funds in Germany.^118^ This argument is supported by literature that correlates the adoption of responsible investment with the size of the fund management industry.^119^

Given the ownership structure of the largest German companies, the following question arises: Is it feasible to introduce a German Stewardship Code in order to promote institutional investors’ engagement with the governance of German investee companies? The answer to this question depends on the domicile of the institutional investors. That can set limits to the geographical scope of a German stewardship Code. Put differently, since many German firms are owned by institutional investors that are predominantly based abroad, any official German stewardship initiative would not have a significant effect on domestic shareholder engagement.

**Fig. 2:**
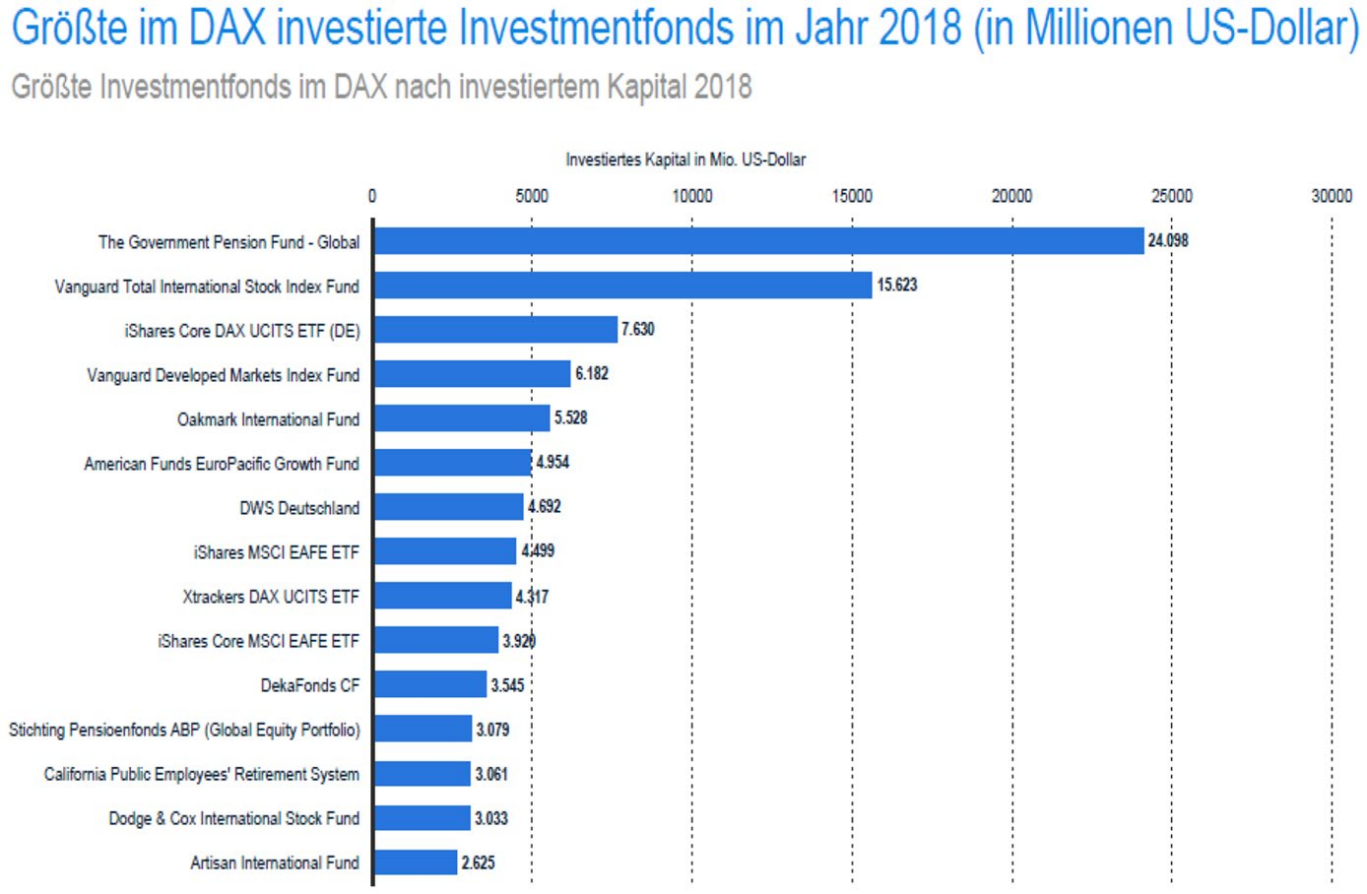
Largest investment funds invested in DAX firms in 2018 (USD million). Source: Statista (2019)

Recent figures show that institutional investors investing in German companies are by far mostly resident outside of Germany. Table 1 and Fig. 2 show the top 15 DAX investors and the 25 largest investment funds that have invested in German DAX companies. According to Table 1, only 10.8% out of the top 34% DAX share can be attributed to Germany-based institutional investors/asset managers. The same picture is illustrated in Fig. 2; only two out of the top 25 funds are based in Germany, and they account for just 16.28% of the investment volume in year 2018. The German asset managers with the largest investment positions in German companies are namely DWS Investment GmbH, Deka Investment GmbH and Allianz Global Investors GmbH.^120^ DWS is the asset management arm of Deutsche Bank^121^; DekaBank^122^ is the investment fund manager for the German *Sparkassen* (savings banks), and Allianz Global Investors^123^ is the asset management arm of Allianz SE. The other fund managers are based either in European countries, such as the Government Pension Fund of Norway, the French Amundi Asset Management SA and the Dutch National Civil Pension Fund, or the United States (Vanguard, Blackrock, Capital One etc.).

**Table 1:** Top 15 investors in DAX firms (2018)

Rank Firm Name	DAX Value in $M Dec-18	% Share DAX Inst	City
1. The Vanguard Group, Inc	32,377.9	4.6%	USA—Malvern, PA
2. BlackRock Fund Advisors	28,026.8	4.0%	USA—San Francisco, CA
3. Norges Bank Investment Management (Norway)	27,638.6	3.9%	NOR—Oslo
4. DWS Investment GmbH	24,104.8	3.4%	DEU—Frankfurt
5. Amundi Asset Management S.A	18,524.6	2.6%	FRA—Paris
6. BlackRock Asset Management (Deutschland) AG	15,103.6	2.2%	DEU—Munich
7. Deka Investment GmbH	13,745.8	2.0%	DEU—Frankfurt
8. Allianz Global Investors GmbH	11,914.5	1.7%	DEU—Frankfurt
9. Harris Associates, L.P	11,522.3	1.6%	USA—Chicago, IL
10. BlackRock Advisors (U.K.), LTD	11,147.8	1.6%	GBR—London
11. Union Investment Privatfonds GmbH	10,700.1	1.5%	DEU—Frankfurt
12. Fidelity Management & Research Company	10,527.2	1.5%	USA—Boston, MA
13. State Street Global Advisors, LTD	9456.5	1.3%	GBR—London
14. Lyxor Asset Management SAS	8806.1	1.3%	FRA—Paris
15. BNP Paribas Asset Management France	8038.0	1.1%	FRA—Paris
Total	241,634.7	34.4%	

Source: adapted from IHS Markit/DIRK (2019), p 11

Based on these figures, it becomes clear that any prospect of effectively regulating the activities of investment fund managers by defining a set of stewardship principles seems to be a challenging task for German policy makers, since the overwhelming majority of the investment decisions in German firms are made by foreign-based vehicles. Certainly, Germany might adopt a voluntary code or a set of guiding principles, but the legislative underpinning of a ‘comply or explain’ rule would not be able to catch any foreign-based institutions. This argument is supported by the geographical scope of application of the existing stewardship codes in other countries.^124^

Consider the UK Code as the paradigm example. At the outset, the UK Code is addressed to ‘institutional investors, by which is meant asset owners and asset managers with equity holdings in UK listed companies’.^125^ That seems to include foreign institutions. It is however important to understand that the application of the UK Code to foreign investors is entirely voluntary. In contrast, the more constraining element of the UK stewardship regime, the ‘comply or explain’ rule,^126^ only applies to domestic UK funds.^127^ This is because the jurisdictional reach of the Financial Conduct Authority (FCA) does not extend to fund managers based outside the UK, who merely invest in shares of companies quoted on the London Stock Exchange, as this will not amount to the carrying out of a ‘regulated activity’ in the UK.^128^ Correspondingly, the ‘comply or explain’ regime has a purely UK focus and cannot apply to foreign investors.^129^ Therefore, the primary focus of the UK Code is on domestically-based institutional investors.

The same would be true in Germany if German regulators chose to adopt a stewardship code. The regulatory authority of BaFin^130^ as the main market watchdog applies to institutional investors domiciled in Germany, and the acquisition of shares in German companies through a foreign investment vehicle does not trigger the application of the relevant investment legislation.^131^ Any German ‘comply or explain’ mechanism that would require institutional investors to respond to stewardship principles could therefore only apply to Germany-based funds or fund managers. This is how the new provisions after the implementation of SRD II apply only to institutional investors and asset managers located in Germany.^132^ The same is true, by the way, for the Corporate Governance Code, where the ‘comply or explain’ principle is enshrined in *Aktiengesetz* § 161, and applies exclusively to companies incorporated under German law.

#### Conclusion

If a stewardship code is not an effective tool, what might be the better alternative to promote the interests of the investee companies, from the perspective of the German government? The obvious answer is to focus any regulatory effort on domestic (target) firms, and to seek improvements here in the form of traditional corporate governance. In other words, since the jurisdictional reach of government regulation over German firms is not a problem, it appears more effective to bundle any regulatory efforts on those. Improving the corporate governance of domestic firms is therefore the politically easier and more effective way of improving engagement, as seen from the perspective of German policy makers. It is in this spirit that the German Corporate Governance Commission has repeatedly revised and updated the German Corporate Governance Code over the past several years, the latest revision of which is from 2019.^133^ It is noteworthy that its latest version, which is yet to come into force, focuses almost exclusively on the operation of both the management board and the supervisory board. Shareholders do not play an important role in corporate governance, German-style.

It is only in the preamble that the German Code states that ‘Institutional investors are of particular importance to companies. They are expected to exercise their ownership rights actively and responsibly, in accordance with transparent principles that also respect the concept of sustainability’.^134^ This statement was introduced in 2017 and clearly mirrors the stewardship idea, albeit in the context of a corporate governance code. In a similar vein, the explanatory notes to the 2019 Code version state that ‘Institutional investors—whether passively managed index funds, active investors or so-called activist investors—are showing increasing interest in corporate governance specifically implemented in the enterprises. Such investors recognise the benefit of standards for good and responsible corporate governance for the performance of their investments; they establish dedicated own ideas regarding corporate governance, and use these as the basis for their voting behaviour in the General Meeting’.^135^

Crucially, neither of these statements carry any serious obligation or other formal requirement; both are rather descriptive declarations that express a certain expectation. This follows the logic developed in this paper: since Germany is virtually unable to regulate any dominant (foreign) institutional investors, it is only able to formulate a number of non-binding statements for them. The substantial principles of the corporate governance code, the recommendations that are subject to the ‘comply or explain’ principle, in essence concern very different topics, mostly about the composition and the obligations of the supervisory board. This is consistent with the rationale developed above: the jurisdictional power of German policymakers fits much better to the domestic players, thus in particular; the board members.

In summary, then, the rationale appears to be that a prudent deployment of government resources leads to public efforts focussing on domestic corporate governance instead of global stewardship.

## Would a German Code Still Make Sense?

The analysis so far has focused on the perspective of the government, and we have seen why policymakers have been so reluctant to adopt an official stewardship code. Still, this does not say anything about the question of whether a stewardship code in the German context would be desirable from a social welfare position.

### Voluntary Compliance

At first, it is important to stress that several arguments speak against the adoption of a German code, thus supporting the German government’s position. One point is that a high number of the (foreign) funds that are active in the German market already comply with a stewardship framework, mostly of foreign origin. There is empirical evidence that they adhere to (foreign) national stewardship codes as well as to international standards of best practice on a voluntary basis. For example, when looking at the top 15 DAX investors, the vast majority of foreign investors (representing 14.1% of the DAX share) annually publish stewardship statements fulfilling their obligations as signatory parties to the UK Stewardship Code.^136^ The high rate of adhering to responsible investment principles on a voluntary basis, despite the lack of a mandatory regulatory framework, has also attracted the interest of academics. Hoepner, Majoch and Zhou^137^ examined the rates of adoption of the UNPRI by asset owners and managers from different jurisdictions. According to their findings, asset managers operating in jurisdictions characterised by soft law regulation^138^ are more willing to (deliberately) adhere to UNPRI than asset managers from jurisdictions with respective mandatory regulation. One factor that could justify the voluntary compliance is the perception that by signalling the ability of the financial industry to regulate itself on a voluntary basis, mandatory regulation by policymakers with their own understanding of responsibility can be avoided. In that way, code creation can be framed as ‘the outcome of a tacit or implicit consensus of institutional actors involved in a self-interested behavioural process’.^139^

UK regulatory authorities aim to achieve a high rate of compliance by foreign investors on a voluntary basis. That is why the 2010 and 2012 versions of the UK Code emphasised that ‘Overseas investors who follow other national or international codes that have similar objectives should not feel the application of the Code duplicates or confuses their responsibilities. Disclosures made in respect of those standards can also be used to demonstrate the extent to which they have complied with the Code’.^140^ The same issue was addressed in the context of the public consultation for the 2010 Code. The FCA expects the UK Stewardship Code 2020 to give foreign owners the proper incentives to sign up to the Code. However, some of the challenges that foreign investors may allegedly face are the different legal and regulatory requirements, different local market conditions, and the need to use local agents across the different jurisdictions.^141^

### Private Initiatives

It is worth noting that in the absence of any governmental scheme, there are a number of private initiatives, mostly from institutional investors, that push for the adoption of a code or even a set of industry principles.

Among them, the most noteworthy are the BVI Rules of Conduct,^142^ a set of principles adopted by the German Investment Funds Association BVI.^143^ BVI is the largest interest group representing the German fund industry, promoting issues of regulation of the fund business as well as business education and competition *vis-à-vis* policy makers and regulators. BVI’s more than 100 members manage assets of some 3 trillion Euros.

The BVI Rules of Conduct, first introduced in 2003, set out a range of voluntary standards and take account of the principle of trusteeship, which places particular obligations on asset managers *vis-à-vis* their investors.^144^ The rules of conduct comprise five principles that asset managers: (1) do not incur undue costs and fees, and do not undermine investor interests through unfair market practices; (2) observe clear execution principles for market-compliant settlement and fair investor treatment; (3) render information in a clear, comprehensive and understandable manner; (4) work towards good corporate governance within the asset management company; and (5) take on social responsibility in ESG.

These principles are subject to a self-defined ‘comply or explain’ rule, that is; the fund companies inform their investors whether, and to what extent, they comply with the rules of conduct. They may deviate from the principles, but then have to disclose this annually and justify any deviations.^145^ The adherence to the BVI’s Rules of Conduct by German asset managers may be unsatisfactory because: (1) there is no legal obligation to disclose compliance or non-compliance with the Rules in contrast to UK asset managers’ obligation to disclose commitment to FRC’s Stewardship Code;^146^ and (2) because there is no monitoring mechanism, such as FRC’s tiering system.

BVI was also among the German stakeholders that responded to the Public Consultation on the EU corporate governance framework. Based on this consultation, the Commission submitted the proposal for the amendment of the Directive 2007/36, which led to the adoption of SRD II. Even though BVI advocated for measures promoting long-termism, shareholders’ communication, and proxy advisors’ transparency, it had a negative position with regard to the regulation of the relationship between asset owners and asset managers. The main argument was the constraints imposed on the contractual freedom of the parties. According to BVI, institutional investors should have the freedom to choose their asset managers and agree with them upon the contractual conditions. There is no need to take additional measures because institutional investors are already obliged to protect their clients’ interests (then AIFM and MiFID I, now UCITS and MiFID II requirements respectively), and they have the means to supervise the affiliated asset managers.

In its comments on the draft ARUG II law^147^ (which implemented the SRD II into German law), BVI welcomed the adoption of transparency requirements for asset managers, but drew attention to their scope of application. More specifically, they were opposed to the application of the ARUG II to Germany-based asset managers in case they undertake the management of assets issued abroad.^148^

The second important instrument to consider is the Code by the German Association of Investment Professionals (DVFA).^149^ DVFA represents more than 1400 financial analysts and asset managers in German financial and capital markets. DVFA supports the professionalization of the investment industry, develops a range of policy standards, and promotes young professionals in the sector. DVFA has recently published its new Stewardship Guidelines, setting out a similar set of issues as the BVI Code.^150^

What to make of such codes? Are they making the need for an official instrument obviated? A careful analysis reveals that a fully-fledged and government-sponsored code such as the UK Stewardship Code is typically much more ambitious than industry self-regulation. That is not very surprising and emerges from several considerations. For example, some observers opine that industry codes are primarily designed to pre-empt any regulation. In other words, they are half-hearted attempts suggesting that self-regulation is in control, but in reality (of course) omitting any requirements that bite.^151^ Another, more charitable, interpretation points to the genesis of such industry-sponsored codes. Industry-made codes often bear the handwriting of consensus: they are typically adopted by a trade association that needs to moderate frequently controversial discussion and thereby needs to take into account many different views and arguments. The result is typically a regulatory standard that is not too restrictive.

In that context, we may wonder why the fund industry is at all concerned with developing de facto regulatory standards, which, in the case of stewardship, are concerned with creating new obligations for (institutional) shareholders rather than to define shareholder entitlements or rights. The seemingly absurd and counterintuitive result is that institutional owners promote their own standards that prescribe costly stewardship activities, thus adding to their own regulatory burden. Apart from the political argument, according to which the industry seeks to fend off a more stringent and prescriptive government-sponsored code, there are at least three reasons that help explain this conundrum.

First, industry self-regulation helps to support market standardisation. The higher the degree of market standardisation, the lower the costs for institutional investors and asset managers to integrate any stewardship policies into their investment strategy. The different standards promoted are de facto replaced or supplemented with a common standard, which may also be more tailored towards the real needs of the industry.^152^ In a similar vein, this may solve the current free-riding situation where some funds are performing engagement policies and thus produce a public good (of better market culture and higher performance) for those that are not.

Secondly, according to Hoepner and others, ‘compliance with voluntary regulation has reputational and socially legitimizing benefits’.^153^ The adoption of stewardship can function more efficiently as a differentiating factor among the compliant and non-compliant market participants, when it is not perceived as compliance with the minimum legal requirements.

Thirdly, having a regulatory framework setting standards of best practice can clarify the legitimacy of institutional investors’ corporate actions. For example, institutional investors face uncertainty with regard to the breach of their fiduciary duties^154^ when they take into consideration non-financial factors in their investment decision-making; or with regard to the breach of ‘acting in concert’ provisions^155^ when they cooperate in corporate governance affairs. A national regulatory initiative backed up by a supervisory authority can give, especially to small-size, institutional investors access to implementation support and know-how sharing networks raising barriers to entry.

### Substitutes

When assessing the merits of adopting regulatory standards on stewardship and shareholder engagement, one must consider any pre-existing substitutes that may—even though in a different form or context—achieve very similar objectives in a different way. Ignoring such substitutes risks overburdening any legal system, and potentially duplicating legal obligations, with the result of dysfunctional rules.

First, it is straightforward and easily understandable that legal critics of any stewardship involvement, as discussed above, will readily find a range of substitutes in the domestic legal system.^156^ As we saw above, these critics will thus argue that the German system of corporate governance, and in particular the supervisory board, is perfectly able to play the same monitoring role that would otherwise be attributed to institutional investors under a stewardship paradigm. With regard to promoting end beneficiaries’ interests, MiFID II and UCITS would thereby function as the appropriate regulatory framework for enhancing transparency between the parties of the investment chain. The existing regulatory framework for financial services providers imposes increased disclosure requirements. The CSR Directive^157^ as well as the Commission’s legislative proposals on sustainable finance promote the integration of long-term considerations in financial institutions’ investment strategy.

SRD II is the most important recent legislative initiative with regard to stewardship because it constitutes mandatory provisions imposing similar disclosure obligations to all EU-based institutional investors and asset managers. Thus, this degree of harmonisation may create a level playing field across Member States.^158^ The EU provisions are minimum legal requirements that can be supplemented by national legislation or codes of conduct.^159^ It is obvious that the UK, as the pioneer in stewardship regulatory initiatives,^160^ will have a competitive advantage against other jurisdictions that have to comply with SRD II, but have not adopted any relative regulation, such as Germany. The German implementation of SRD II stays rather close to the text of the European Directive and does not develop these requirements into a workable framework for practical application.^161^

All these arguments would however rest on the implicit assumption that the German legal framework for shareholder engagement or corporate monitoring is de facto effective in curbing managerial slack. In other words, the argument that powerful substitutes exist in German law that justify the absence of a stewardship code is only acceptable if those substitutes are equally effective as a fully-fledged stewardship code would be. This, however, is very much an open question. The effectiveness of the monitoring procedures mandated by German law have long been questioned.^162^ For example, it is unclear whether German supervisory boards are sufficiently independent to exercise an adequate review of managerial actions.^163^ Equally, the shortcomings of German group law (*Konzernrecht*) have long been documented, and only very few other countries have followed this example.^164^

A second substitute may be found in the self-commitment by some institutional investors, in particular those who are members of either or both of the associations BVI and DVFA.^165^ However, we saw above that these self-binding codes do not live up to the obligations of a sophisticated stewardship code, and do not come with a comparable legal force. In fact, such market initiatives will frequently be adopted with the specific objective of fending off any more stringent legal action.

Institutional investors and asset managers can also self-commit to either national or international standards of best practice. Among the most prominent international standards are the ICGN Global Stewardship Principles and the UNPRI. The international scope of these principles makes the choice of complying with them superior to the choice of complying with national codes of conduct. This is especially the case for jurisdictions that have so far not developed a regulatory framework for stewardship. Based on this, the marginal benefits from introducing a stewardship code in Germany may be small, provided that there is a body of international principles with a broader base of signatory parties.^166^

We should also take into consideration that the introduction of stewardship regulation on the national level would require resources for the establishment of the necessary monitoring and enforcement infrastructure. The benefits expected from such an initiative would have to exceed the administrative cost of enforcement, and such an estimation should take into consideration the inefficiencies of the existing monitoring mechanisms.^167^

### Discussion

Can the problems concerning the applicability of a national Stewardship Code to foreign owners and the existence of private initiatives as well as possible substitutes justify the constant refusal of the German government to follow the trend and to set up a UK-style stewardship code? There are several factors that would still give ample room for a German code to play a useful role in the German corporate landscape.

First, the sceptics’ position would be understandable if the sole objective of a code was to promote the interests of the investee companies. However, as we saw above, it is among the primary objectives of any legislation including the UK Code and SRD II to also further the interests of end beneficiaries. Their interests can be efficiently protected through achieving greater transparency between the parties of the investment chain, asset managers-institutional investors, and institutional investors-end beneficiaries.^168^ Greater transparency is perceived as an important condition for making effective stewardship a differentiating factor across the firms of the investment management industry,^169^ a market with strong anti-competitive forces.^170^ An improvement of competition is in the interests of institutional investors’ and asset managers’ clients and, thus, beneficial for market quality and integrity.^171^ Therefore, even if many institutional investors are not based in Germany, their activity affects German companies and German end beneficiaries. With the aim to promote the interests of the latter stakeholders, a German stewardship initiative would be both politically feasible and legitimate.

**Fig. 3:**
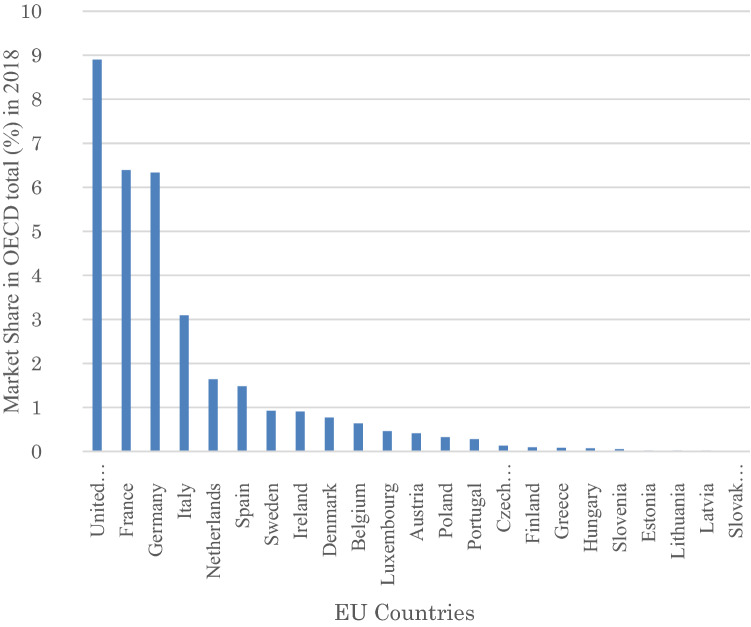
Market share of insurance industry in EU countries as part of OECD total (2018). Source: OECD, https://stats.oecd.org/Index.aspx?DataSetCode=INSIND

**Fig. 4:**
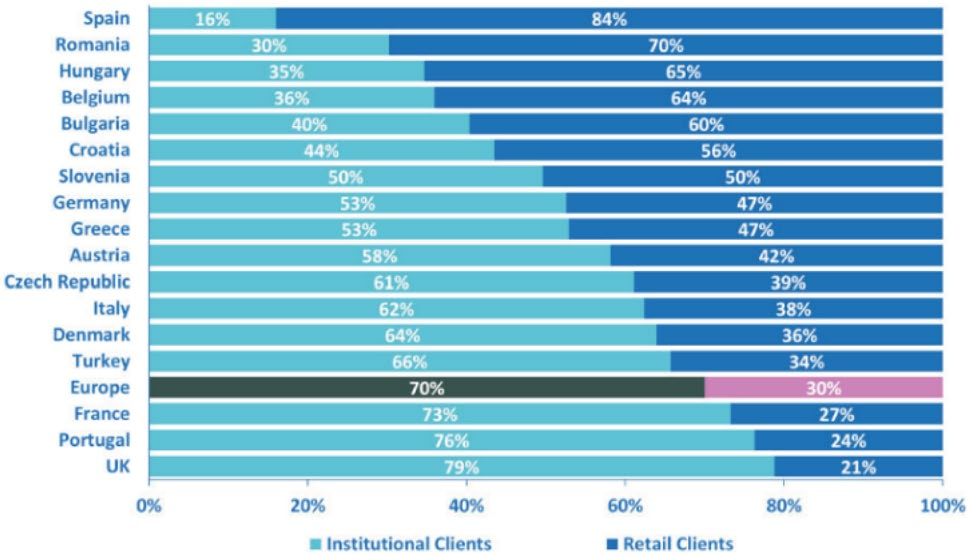
AuM by type of client and country at end of 2017. Source: EFAMA (2019), p 5

Secondly, a stewardship initiative may be even more urgent in Germany than in other European jurisdictions. The reason is the clientele of German asset managers. In Germany, the pension funds industry is not as developed as in the UK or the Netherlands (see Table 2). This is also the case for the insurance industry, where the UK and France are the leading insurance markets (Fig. 3). German asset managers’ clients are split equally into institutional and retail investors, in contrast to the UK and France, where more than 75% of asset managers’ clients are institutional investors (Fig. 4).

**Table 2:** Number of pension funds and their assets under management (2018)

Country	Number of pension funds	Assets held by pension funds (billion EURO)	Number of members	Number of beneficiaries
Netherlands	260	1360.15	5,646,763	13,046,483
UK	1300	1173.80	20,000,000	10,493,000
Switzerland	1650	749.06	4,174,580	1,183,910
Germany	171	184.80	7,903,000	1,493,000
Ireland	71,340	147.60	437,711	750,000
Italy	252	111.81	4,034,220	116,282
Spain*	1576	76.47	4,583,652	97,551
Sweden*	62	36.72	1,112,062	187,637
Norway	84	34.80	148,000	360,000
Iceland	24	28,47	264,902	126,222
Austria	10	22,70	928,000	99,000
Belgium	197	32.00	974,842	759,473
Portugal	189	18.43	166,530	131,831
France	25,489	15.90	2,400,000	n/k
Croatia	12	12.23	1,844,272	n/k
Romania	7	8.53	7,042,179	20,000
Bulgaria	18	5.97	3,965,174	n/k
Finland	47	4.33	166,530	48,796
Estonia	22	3.60	744,675	37,373
Luxembourg	13	1.55	16,466	n/k
Hungary	4	0.77	n/k	n/k
Total	101,437	4028.21		

Source: PensionsEurope (2018), pp 4, 7

Instead, retail clients usually invest in investment funds, while institutional investors delegate discretionary mandates to their asset managers. One of the main differences between the two investment practices is that, in the case of discretionary mandates, the investment strategy is agreed beforehand with each client, so that they are tailor-made to the specific investment goals of each individual investor.^172^ In Germany, the main investment vehicle for both retail and institutional investors is the investment fund.^173^ The investment strategy of the investment fund is predetermined, and clients can choose to either opt in or to not invest in it at all. The fact that end beneficiaries do not have a say in the investment strategy of the fund makes their support at the stage of selecting and monitoring the fund even more important. Enhanced transparency achieved by imposing disclosure requirements with regard to stewardship can support end beneficiaries make selection decisions and monitoring on an informed basis.^174^ End beneficiaries should then be able to evaluate if a fund’s strategy aligns with their investment preferences.^175^

**Fig. 5:**
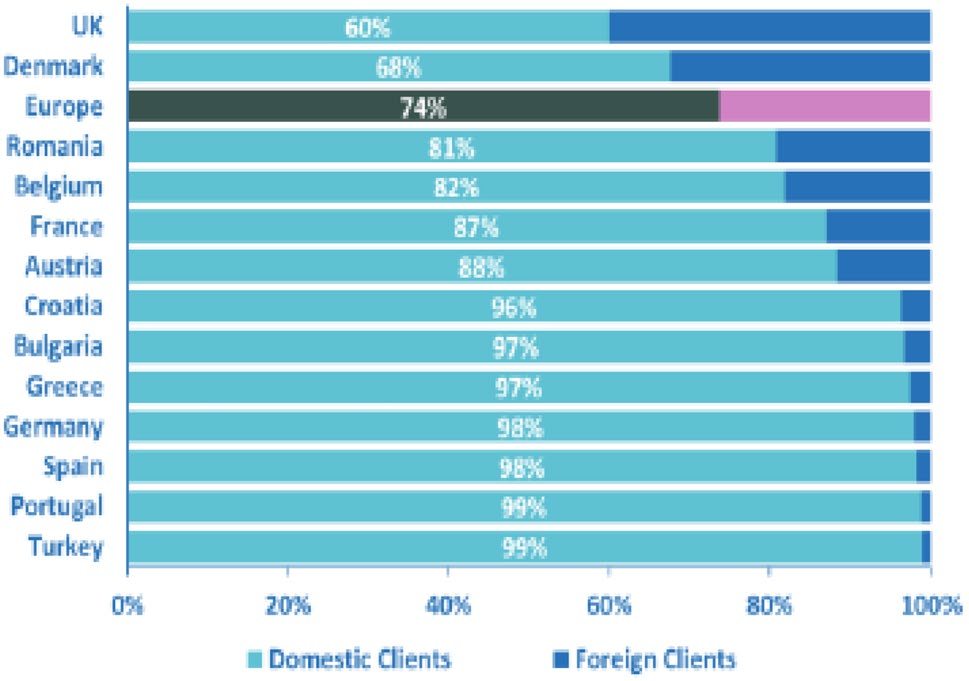
Domestic and Foreign Clients at end 2017. Source: EFAMA (2019), p 6

True, the main problem concerning the possibility of regulating the activities of foreign institutional investors through German regulatory initiatives remains, but is less salient with regard to end beneficiaries than investee companies. The reason lies in the ‘home bias’ that is typically inherent in the process of selecting asset managers (see Fig. 5). Based on this bias, German end beneficiaries will tend to frequently select German funds to manage their assets, so that any legislative initiative that improves the activities of Germany-based funds will disproportionately benefit German institutional and retail investors.^176^

As we saw above, the largest investors in DAX companies, both foreign and German, already have adopted some type of a stewardship agenda in their investment strategy. This may be based on a foreign national code or on domestic or international standards of best practice, typically on a voluntary basis. Should any German stewardship initiative be forthcoming, it would naturally be more effective in addressing domestic small and medium size institutional investors.^177^ A national regulatory framework for stewardship, even if it does technically not bind foreign investors, may still support domestic institutional investors or end beneficiaries in their selection of domestic asset managers and institutional investors respectively. If stewardship is compatible with end beneficiaries’ preferences, and if there is an efficient mechanism of monitoring stewardship activities, the adoption of stewardship regulation can give a competitive advantage to institutional investors and asset managers that adopt stewardship activities either on a mandatory or on a voluntary basis.^178^

Further benefits from a German code may be expected in less salient areas. For example, a German code may simply define expectations of what policy makers believe good stewards should do, even though enforcement powers may be limited. It would equally standardise market practices, which are currently, as we have seen, relying on a patchwork of foreign and international standards as well as domestic industry initiatives. It would clarify the obligations and reporting requirements of investee companies and define legally acceptable practices, such as to what extent ‘acting in concert’ is permissible. In sum, a stewardship code would contribute to improving the market culture of the German capital market, essentially a public good that requires a neutral, government-led approach.^179^

## Conclusion

This paper has explored the role of shareholder engagement and stewardship in the German context. Although shareholders have taken a more assertive role as active investors recently, it is surprising to see the comparative reluctance of German policy makers towards promoting a stewardship code that would define and promote engagement practices. Thereby, Germany is swimming upstream by refusing to follow the international trend towards such a code of best practices.

While many doctrinal and functional arguments are advanced to explain this refusal, this paper has argued that the main reason for Germany’s reluctance may be rooted in politics: simply put, the comparatively small size of the German fund industry relative to the economy may explain why regulating it may not be the government’s top priority. Instead, the German position is following traditional patterns of domestic corporate governance and law.

Still, I argue that a stewardship code would be a useful complementary instrument for the German market. It would standardise market practices, improve market culture, and clarify the obligations of investee companies. Most importantly, however, it would benefit domestic investors that are typically ‘home biased’ and thereby frequently disproportionately invested in domestic funds.

The implementation of SRD II into German law should therefore not be seen as the end of the debate. Rather, we should strive to continue our efforts towards strengthening engagement and accountability in the German market.

## Footnotes

1 See, for example, Chiu (2013).

2 Directive (EU) 2017/828 of the European Parliament and of the Council of 17 May 2017 amending Directive 2007/36/EC as regards the encouragement of long-term shareholder engagement [2017] OJ L132/1 (SRD II).

3 Cheffins (2010), pp 1009–1010; Reisberg (2015), pp 220–221.

4 Walker (2009), para. 5.10.

5 Ibid., para. 5.11.

6 Myners (2009), para. 38.

7 Kay (2012).

8 The Walker Review (Walker 2009) had recommended that the FRC’s remit should be extended in this way.

9 See Financial Reporting Council (2010a).

10 See Financial Reporting Council (2010c).

11 Financial Reporting Council (2010b).

12 Financial Reporting Council (2012).

13 The most recent version of the UK Stewardship Code came into force on 1 January 2020. See Financial Reporting Council (2020).

14 On these two sides of stewardship, see Katelouzou (2019).

15 Financial Reporting Council (2019a), para. 1.11.

16 Now the Financial Conduct Authority (FCA).

17 FCA Handbook, Conduct of Business Sourcebook (COBS) 2.2.3R (06/12/2010) and 2.2A.5R (03/01/2018).

18 European Commission (2010a).

19 European Commission (2010b), s. 4.1.

20 European Commission (2010a), s. 3.5.

21 Barnier (2010).

22 European Commission (2010c).

23 This resulted in another, broader green paper: European Commission (2011).

24 Antunes et al. (2011), s. 3.1.4.

25 European Commission (2012).

26 European Commission (2012), p 5.

27 See Hopt (2015), pp 176 et seq.

28 SRD II (n. 2), Art. 2(1).

29 A database is maintained by the European Corporate Governance Institute at https://ecgi.global/content/codes-stewardship?field_categories_tid=Stewardship. For a good discussion, see Hannigan (2017).

30 International Corporate Governance Network (2016), ICGN Global Governance Principles.

31 OECD (2011a).

32 OECD (2015), G20/OECD Principles of Corporate Governance. See in particular section III. An explicit reference to the stewardship idea is at pp 29–30.

33 European Fund and Asset Management Association (2018). The 2018 EFAMA Code is based on its predecessor, the 2011 EFAMA Code for External Governance.

34 The EU adopted a ‘CSR Directive’ in 2014 to introduce so-called ‘non-financial reporting’. See Directive 2014/95/EU of the European Parliament and of the Council of 22 October 2014 amending Directive 2013/34/EU as regards disclosure of non-financial and diversity information by certain large undertakings and groups [2014] OJ L330/1.

35 See https://www.unpri.org/.

36 Scott (2012).

37 European Commission (2018a).

38 European Commission (2018b).

39 Financial Reporting Council (2020). Financial Reporting Council (2019b).

40 International Corporate Governance Network (2019).

41 See above Sect. 2.1.

42 International Corporate Governance Network (2019), p 2.

43 See Kahan and Rock (2014); see also Pargendler (2016).

44 See e.g. Ringe (2013). For an international comparison, see Puchniak, Baum and Nottage (2017).

45 Bhagat and Black (1999); Bhagat and Black (2002); Fernandes (2008).

46 See Fisch (2020).

47 Fisch, Hamdani and Davidoff Solomon (2019), p 37.

48 Appel, Gormley and Keim (2016).

49 Myners (2001), paras. 5.34, 5.88; Arsalidou (2012), p 364; Reisberg (2015), pp 231, 247; Heinen (2019), pp 20, 27; Alvaro, Maugeri and Strampelli (2019).

50 Bebchuk and Hirst (2019a), p 2057.

51 Walker Review (Walker 2009), paras. 5.9, 5.16; European Commission (2010a); Financial Conduct Authority/Financial Reporting Council (2019), paras. 2.13, 5.6, 5.18.

52 Böckli et al. (2015). The European Company Law Expert Group Response to the Public Consultation for Commission’s Proposal for a Directive of the European Parliament and of the Council amending Directive 2007/36/EC as regards the encouragement of long-term shareholder engagement and Directive 2013/34/EU as regards certain elements of the corporate governance statement.

53 Opinion Letters of Aberdeen Asset Management, CFA Institute, GC100, ICAEW, ICGN, JP Morgan Asset Management, NAPF to Consultation on a Stewardship Code for Institutional Investors 2010.

54 Arsalidou (2012), pp 346, 356; OECD (2015), G20/OECD Principles.

55 Opinion Letters of Alex Edmans, Allianz Global Investors, Association of Member Nominated Trustees, CFA Society UK, GC100, Black Sun plc, Implementation Taskforce, M&G Investments, Merian Global Investors, Share Action and Smart Pension to Public Consultation for FRC’s Proposed Revision to the UK Stewardship Code, all available at https://www.frc.org.uk/consultation-list/2019/consulting-on-a-revised-uk-stewardship-code.

56 Financial Conduct Authority (2019a), para. 2.20.

57 Chiu (2013).

58 Financial Reporting Council (2019a), para. 2.20.

59 See Kahan and Rock (2014).

60 Cox, Brammer and Millington (2004); Jansson and Biel (2011); Bauer, Ruof and Smeets (2019).

61 Bebchuk and Hirst (2019a).

62 Hawley and Williams (2007); Bebchuk and Hirst (2019b).

63 Sievänen, Rita and Scholtens (2012).

64 Financial Reporting Council (2010a).

65 Bebchuk and Hirst (2019b).

66 Ringe (2015).

67 For EU-companies: Observatoire de l’Epargne Européenne and INSEAD OEE Data Services (2013); for German companies: OECD (2011a), p 115.

68 See below Sect. 3.2.4.

69 OECD (2011a), p 112; Rapp and Strenger (2015), pp 20–22.

70 Kalemli-Özcan et al. (2013).

71 IHS Markit/DIRK (2019).

72 The *Aktiengesetz* ist available in English at https://www.nortonrosefulbright.com/en/knowledge/publications/bc19a262/german-stock-corporation-act-aktiengesetz.

73 For the distinction between activism, engagement and stewardship see Alvaro, Maugeri and Strampelli (2019).

74 For the period 1998–2010: Mendoza, van der Elst and Vermeulen (2010); for the period 2010–2013: Lafarre (2014).

75 Engert (2019); Moeser (2019).

76 Gantchev (2013).

77 Financial Reporting Council (2020).

78 See Köhler et al. (2019).

79 Berger (2019).

80 More than 55% of the shareholders voted in favour of a ‘no-confidence’ motion. See Chazan (2019).

81 Berger (2019), p 86.

82 For example, the voting behaviour of Allianz Global Investors in Germany—which is available on the website—over the twelve-month period from October 2017 to September 2018 shows that the highest rejection rate (out of a total of 50%) can be found in the agenda for Executive Board compensation.

83 Chazan (2019).

84 See, in particular, Hommelhoff (2015), pp 1332, 1335.

85 Fleischer and Strothotte (2011), p 227.

86 *Aktiengesetz* § 76(1).

87 *Aktiengesetz* § 111(1). See Hell (2019), p 342.

88 Fleischer (2011), p 166.

89 *Aktiengesetz* § 53a.

90 Fleischer and Strothotte (2011), p 228.

91 Fleischer and Strothotte (2011), fn. 69; see also Fleischer (2009), p 524.

92 Forstmoser (2005), p 803.

93 Fleischer and Strothotte (2011), p 227.

94 Fleischer (2011), p 167.

95 Lutter (1988), § 54, para. 2.

96 Fleischer (2011), p 167.

97 This view has a rich tradition, interestingly, in UK company law. See from the seminal case law *Pender v. Lushington* (1877) 6 ChD 70, 75; *North-West Transportation Co Ltd v. Beatty* [1887] UKPC 39, (1887) 12 App.Cas. 589; *Burland v. Earle* [1902] AC 83 (PC); *Carruth v. Imperial Chemical Industries Ltd* [1937] AC 707 (HL); *Peter’s American Delicacy Co Ltd Heath* [1939] HCA 2, (1939) 61 CLR 457, 504; *Northern Counties Securities Ltd v. Jackson & Steeple Ltd* [1974] 1 WLR 1133, 1144. See in more detail Ringe (2017), pp 265–267.

98 See e.g. Hirt, Hopt and Mattheus (2016); Vetter (2016); Bachmann (2017); Hopt (2017); Hein (2018), pp 239–290.

99 Schneider (2012), pp 530–531.

100 Regierungskommission Deutscher Corporate Governance Kodex (2017), German Corporate Governance Code (7 February 2017).

101 Bassen et al. (2016).

102 Aguilera and Crespi-Cladera (2016), pp 50–57.

103 Gilson (2006), p 1651.

104 See e.g. Johnson et al. (2000).

105 See Ringe (2015).

106 Ringe (2015), pp 526 et seq.

107 See e.g. Chiu (2013); Chiu (2014); Hannigan (2017).

108 Skypala (2012).

109 Kingman (2018).

110 Ibid., p 46 (recommendation 42).

111 Rock (2018), pp 375 et seq.

112 Ibid., p 379.

113 Hoepner (2019).

114 Dimson et al. (2015). See also Barko et al. (2017).

115 Aghion et al. (2013).

116 Schmidt and Fahlenbrach (2017).

117 James et al. (2018).

118 OECD (2011b), p 106.

119 Scholtens and Sievänen (2013).

120 One additional factor distinguishing the German asset management market from the UK one is the fact that almost half of the German asset managers are part of the banking groups, while 80% of the UK asset managers operate as stand-alone companies. See European Fund and Asset Management Association (EFAMA) (2019), p 9.

121 DWS is a signatory party to the UK Stewardship Code and is categorized in Tier 1 of asset managers. It is also a signatory party to the UNPRI since 2008. DWS has published two Responsible Investment Statements (January 2017 and July 2018). In these statements DWS refers explicitly to stewardship as part of its ‘active ownership’ philosophy and expresses its ‘aim to comply with and to assist (its) clients in complying with local stewardship codes given the increasing pace and scope of regulation’. See DWS (2018), paras. 2 and 4.

122 Deka Investment GmbH is not a signatory party to the UK Stewardship Code but it has adhered to the UNPRI since 2010.

123 Allianz GI is a signatory party to both the UK Stewardship Code as a Tier 1 asset manager and the UNPRI since 2007. It has participated repeatedly in the FRC public consultation related to amendments of the UK Stewardship Code.

124 Walker Review (Walker 2009), para. 6.4; Cheffins (2010), pp 1004 et seq.; Arsalidou (2012), pp 355 et seq.; Reisberg (2015), pp 236–238.

125 Financial Reporting Council (2012), p 2.

126 COBS 2.2.3R.

127 See Financial Services and Markets Act 2000, s. 418; PERG 2.4.5 (01/07/2005).

128 Davies (2015), p 376. See also Cheffins (2010), p 1015.

129 Initially, the UK Code was introduced without a corresponding ‘comply or explain’ requirement. Instead, the FRC simply encouraged ‘all institutional investors to report if and how they applied the Code’ (Financial Reporting Council (2010b), para. 21). At the same time, however, the FRC stated that it ‘hope(s) that investors outside the UK will commit to the Code’ but recognises ‘that, in practice, local institutions usually take the lead in engagement’ (ibid., para. 25).

130 *Bundesanstalt für Finanzdienstleistungsaufsicht*.

131 Most notably, the *Kapitalanlagegesetzbuch* (KAGB) [German Capital Investment Act].

132 For a critical perspective, see Baums (2019), p 608; Tröger (2019). See also Koch (2020), p 3.

133 The 2019 revision of the Code (Regierungskommission Deutscher Corporate Governance Kodex (2019a), German Corporate Governance Code 2019 (9 May 2019)) came into force on 20 March 2020.

134 Regierungskommission Deutscher Corporate Governance Kodex (2017), Foreword, para. 3.

135 Regierungskommission Deutscher Corporate Governance Kodex (2019b), German Corporate Governance Code as resolved by the Commission (Code including rationale, 9 May 2019).

136 A list of signatories of the UK Stewardship Code is available (Financial Reporting Council, ‘Tiering of 2012 Stewardship Code Signatories’, https://www.frc.org.uk/investors/uk-stewardship-code/uk-stewardship-code-statements (accessed 6 Jan 2020). See also above nn. 121, 122, 123.

137 Hoepner (2019).

138 ESG regulation has been used in their analysis as a proxy of the regulatory framework of responsible investment. Thus, the analysis has not taken into consideration any specific stewardship regulatory initiative.

139 Haxhi et al. (2013), p 543.

140 Financial Reporting Council (2012), p 3.

141 Financial Conduct Authority (2019c), para. 3.32.

142 BVI (2019).

143 Bundesverband Investment und Asset Management e.V. (BVI).

144 The current version of the BVI Rules of Conduct has been in force since 1 July 2019.

145 However, BVI states that ‘to the extent that individual rules are not applicable in view of the respective investment strategy (such as benchmark-oriented investing or property investments), business activity or organisational structure, deviations do not need to be explained separately’ (BVI (2019), p 2).

146 COBS 2.2.3.

147 *Gesetz zur Umsetzung der zweiten Aktionärsrechterichtlinie (ARUG II)* of 12 December 2019, *Bundesgesetzblatt* [Federal Gazette] 2019 I, p 2637.

148 ‘Wir begrüßen, dass die Regelung als Anknüpfungspunkt für die Pflichten von Vermögensverwaltern deren Zulassung im Inland vorsieht (s.u. zu § 134c Abs. 4 AktG-E). Wir teilen jedoch nicht die Auffassung, dass die Pflichten auch Anwendung finden, soweit es sich um Aktien handelt, die im außereuropäischen Ausland notiert sind’. See BVI (2018).

149 Deutsche Vereinigung für Finanzanalyse und Asset Management e.V. (DVFA).

150 DVFA (2020).

151 Haxhi, van Ees and Sorge (2013). See also Puchniak and Tang (2019), who coin the term ‘preemptive corporate governance’ for this.

152 Binder and Gutzwiller (2013).

153 Hoepner et al. (2019), p 14.

154 Juravle and Lewis (2008); Sandberg (2010); Sandberg (2013).

155 Fleischer and Strothotte (2011), p 226.

156 See above Sect. 3.2.1.

157 See above n. 34.

158 European Commission (2014), pp 6–7.

159 Financial Conduct Authority/Financial Reporting Council (2019), paras. 1.6, 6.9, 6.13; Financial Conduct Authority (2019c), para. 3.86.

160 Juravle and Lewis (2008).

161 Koch (2020); Tröger (2019).

162 See e.g. OECD (2011a), p 119.

163 Baums and Scott (2005); Heidrick and Struggles (2011).

164 Antunes et al. (2011), p 59.

165 See above Sect. 4.2.

166 A list of ICGN Global Stewardship Principles endorsers is available at https://www.icgn.org/policy/icgn-global-stewardship-principles-endorsers (accessed 6 Jan 2020), and a list of UNPRI signatories is available at https://d8g8t13e9vf2o.cloudfront.net/Uploads/w/j/y/signatorydirectoryupdated112019_169996_778605_873356.xlsx (accessed 6 Jan 2020).

167 Sergakis (2019); Chiu (2019).

168 European Commission (2014).

169 Financial Conduct Authority (2019d), paras. 1.13–1.15.

170 Financial Conduct Authority (2019b).

171 Financial Conduct Authority (2019c), para. 1.1.

172 European Fund and Asset Management Association (2019), p 3.

173 Ibid., p 6.

174 FairPensions (2010).

175 See text accompanying n. 60 above.

176 Bauer et al. (2013).

177 Qualitative analysis of the responses of UK stakeholders in the context of the public consultation for the UK Stewardship Code 2010 has showed that medium and small-size asset managers were reluctant towards the introduction of the Code basically because of the costs to be borne with regard to the fulfilment of stewardship obligations. Some of these asset managers were namely F&C Investments, M&G Investments, Rathbone Investment Management, Rothschild Wealth Management (UK) Ltd, Royal London Asset Management, South Yorkshire Pensions Authority. Most of the initially reluctant asset managers have since been categorized as Tier 1 asset managers.

178 According to a survey by CREATE-Research/DWS, stewardship is a central competition factor for passive investment funds (Rajan 2019).

179 For a similar conclusion, see Baums (2019), p 615.
